# Depletion of ribosomal protein S19 causes a reduction of rRNA synthesis

**DOI:** 10.1038/srep35026

**Published:** 2016-10-13

**Authors:** Giada Juli, Angelo Gismondi, Valentina Monteleone, Sara Caldarola, Valentina Iadevaia, Anna Aspesi, Irma Dianzani, Christopher G. Proud, Fabrizio Loreni

**Affiliations:** 1 Department of Biology, University of Rome Tor Vergata, Roma, Italy; 2 Centre for Biological Sciences, University of Southampton, Southampton, UK; 3 Department of Health Sciences, Università del Piemonte Orientale, Novara, Italy

## Abstract

Ribosome biogenesis plays key roles in cell growth by providing increased capacity for protein synthesis. It requires coordinated production of ribosomal proteins (RP) and ribosomal RNA (rRNA), including the processing of the latter. Here, we show that, the depletion of RPS19 causes a reduction of rRNA synthesis in cell lines of both erythroid and non-erythroid origin. A similar effect is observed upon depletion of RPS6 or RPL11. The deficiency of RPS19 does not alter the stability of rRNA, but instead leads to an inhibition of RNA Polymerase I (Pol I) activity. In fact, results of nuclear run-on assays and ChIP experiments show that association of Pol I with the rRNA gene is reduced in RPS19-depleted cells. The phosphorylation of three known regulators of Pol I, CDK2, AKT and AMPK, is altered during ribosomal stress and could be involved in the observed downregulation. Finally, RNA from patients with Diamond Blackfan Anemia (DBA), shows, on average, a lower level of 47S precursor. This indicates that inhibition of rRNA synthesis could be one of the molecular alterations at the basis of DBA.

Ribosome biogenesis is an energetically expensive process that occurs in the nucleolus and is tightly regulated according to cell growth conditions. It requires the activity of all three nuclear RNA polymerases (Pol), each of them dedicated to the transcription of a different ribosomal components^1^. Specifically, Pol II produces ribosomal protein mRNAs that are transported in the cytoplasm and translated into ribosomal proteins (RPs) according to cell growth rate^2^,^3^. Pol III synthesizes the 5S ribosomal RNA (rRNA), and Pol I is dedicated to the synthesis of the other rRNAs. Primary product of Pol I transcription is 47S pre-rRNA (also called 45S), which includes 18S, 5.8S and 28S rRNAs^4^. Once transcribed, 47S pre-rRNA is cleaved at specific sites (endo- and exo-nucleolitic cleavages) by RNAses to produce a series of characteristic intermediates (45S’, 41S, 32S, 30S, 21S, 12S), and finally, the mature rRNA species^4^,^5^,^6^. Pol I activity accounts for up to 60% of transcription in a eukaryotic cell^7^. It requires a number of accessory factors, which facilitate polymerase recruitment, initiation, promoter escape, elongation and termination^1^.

The transcription of rDNA is dependent on three basal transcription factors: the “selectivity complex” (SL1 or TIF-IB), the HMG1 box architectural upstream binding factor (UBF) and the Pol I. Both UBF and SL1 make direct contacts with two sequence elements known as the upstream control element (UCE) and the core. In the case of UBF, it probably binds to these promoter elements as a dimer. UBF is thought to bind the promoter first, enabling the subsequent recruitment of SL1 and Pol I. The initiation factor TIF-IA is also part of this complex^5^,^8^,^9^. In addition, many accessory factors are now known to assist at each stage of this transcription cycle, some of which allow the integration of transcriptional activity with metabolic demands^1^. Stringent regulatory mechanisms presumably operate to ensure a precise balance between the requirement for, and the availability of, rRNA. This is necessary to allow the cells to control their capacity for protein synthesis in response to changing metabolic needs, linked, e.g., to cell growth and proliferation. In fact, transcription by Pol I is low when nutrients or mitogens are limiting, but is enhanced when the availability of these growth stimuli increases. In addition, Pol I transcription is regulated in response to a range of cellular stresses and during many fundamental cellular processes, such as cellular differentiation and cell cycle^5^,^10^,^11^,^12^. A range of cellular control pathways has been shown to mediate such acute changes in rRNA synthesis. Final effectors of these pathways directly regulate the activity of the Pol I transcription machinery, either positively or negatively^5^,^9^,^12^.

Interest in mammalian ribosome biosynthesis has been stimulated by its involvement in the pathogenesis of a number of inherited and acquired diseases including cancer^13^,^14^. Mutations in genes encoding components of the ribosome or proteins involved in ribosome maturation, assembly, or export have been identified as the cause of a variety of clinical symptoms^13^. For instance, Diamond Blackfan Anemia (DBA), characterized by a reduced number of erythroid precursors, is caused by mutations in one of several genetic loci which code for RPs of both small and large ribosomal subunit^13^,^15^,^16^.

Most RPs interact with rRNA and might function as RNA chaperones, not only during the assembly of the ribosome particle, but also in the stabilization of important domains of the rRNA during its maturation^4^. RP depletion, with a few exceptions, leads to the accumulation of specific rRNA precursors, highlighting their individual role in specific steps of the rRNA processing^17^,^18^. For instance, it has been shown that the depletion of RPS19 in human cell lines by siRNA causes decreased ribosome synthesis with accumulation of rRNA 21S^17^,^18^,^19^.

In this study, we aimed to address the effect of RPS19 deficiency on rRNA synthesis. We found that the depletion of RPS19 causes a reduction of 47S rRNA in a number of cell lines of different origins. The lower level of rRNA precursors reflects decreased recruitment of Pol I onto the rDNA promoter. Moreover, the analysis of a number of RNA samples from DBA patients shows, on average, a lower level of 47S rRNA with respect to control samples. This suggests that the molecular alterations that occur in DBA cells include a reduction in rRNA synthesis.

## Results

### Depletion of RPs causes a decrease of the 47S rRNA level

**Figure 1:**
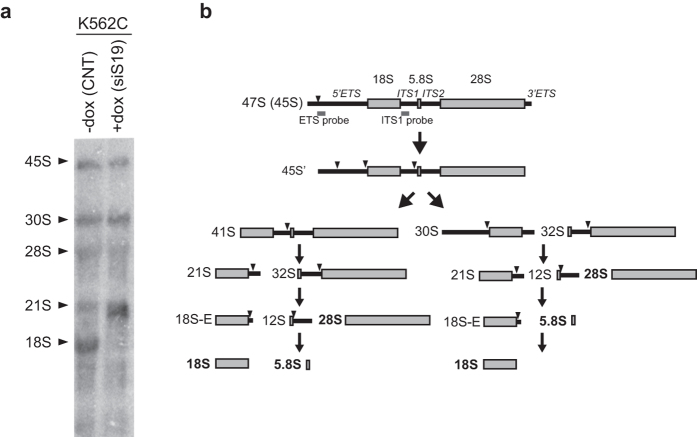
rRNA maturation during RPS19 depletion. (**a**) Total RNA was extracted from K562C cells, untreated (−dox) or incubated with doxycycline for 4 days to induce an siRNA against the S19 mRNA (+dox) and analyzed by Northern blot with a probe for the ITS1 region. The figure shows a PhosphorImager image with major rRNA species indicated by black arrow points. (**b**) Scheme of rRNA processing pathways in mammals. The name of rRNA precursors and major cleavage sites are indicated. The approximate position of the probe is shown within the ITS1.

**Figure 2:**
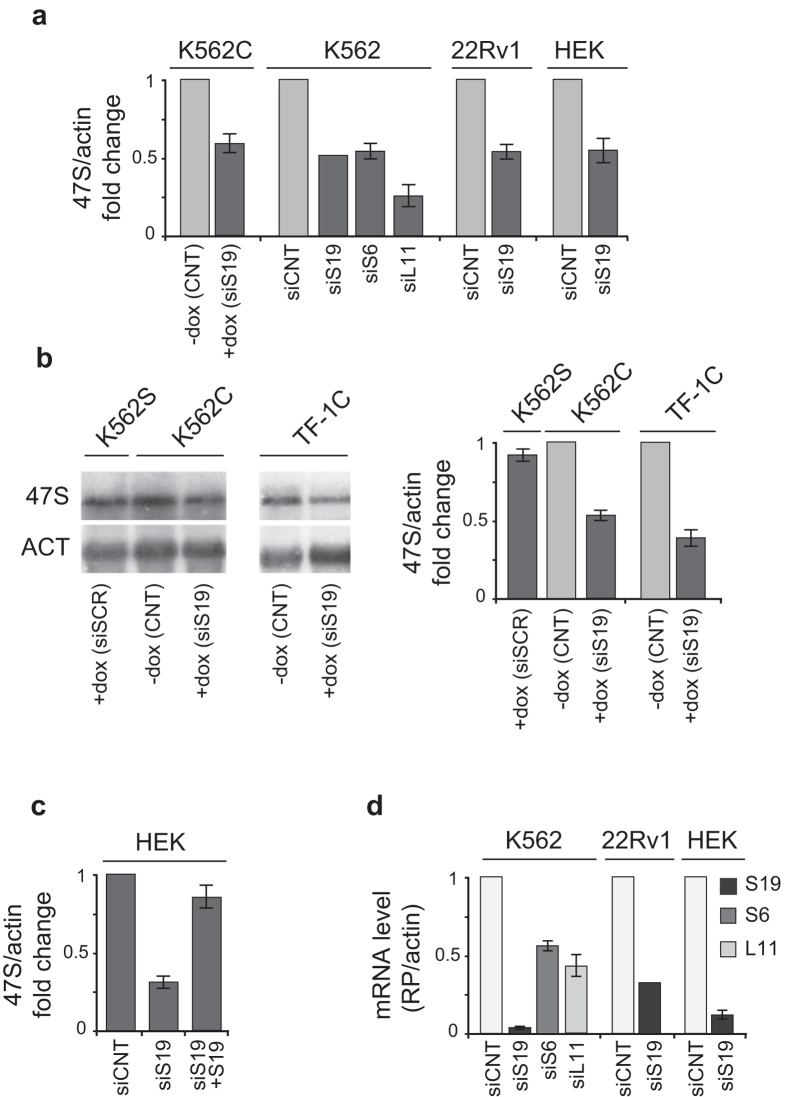
Analysis of 47S pre-rRNA level. (**a**) K562C cells were incubated with vehicle (−dox) or with doxycycline for 4 days (+dox) whereas K562, 22RV1 and HEK293 cells were transfected with unrelated siRNA (siCNT) or siRNA specific for RPS19 (siS19), RPS6 (siS6) or RPL11 (siL11). Total RNA was analyzed by RT-qPCR with primers specific for 5′ETS region of 47S rRNA and β-actin. The results of triplicate RT-qPCR from three independent RNA preparations are reported as a column plot of the mean ± s.e.m. of 47S rRNA/actin mRNA (siS19 is from a single triplicate experiment). (**b**) Total RNA extracted from K562S cells incubated with doxycycline for 4 days to induce a scrambled siRNA (+dox), from K562C or from TF-1C cells, untreated (−dox) or incubated with doxycycline for 4 days to induce an siRNA against the S19 mRNA (+dox) was analyzed by Northern blot with a 5′ETS oligonucleotide probe (indicated in Fig. 1b). An example of Northern blot is shown on the left. Uncropped original images are shown in Supplementary information. The results from three independent RNA preparations are reported as a column plot of the mean ± s.e.m. of 47S rRNA/actin mRNA. (**c**) HEK293 cells were transfected with control (siCNT) or RPS19 specific siRNA (siS19). Part of siS19 cells were then transfected with an RPS19 overexpressing plasmid (siS19 + S19). RNA was analyzed by RT-qPCR with primers specific for 5′ETS region of 47S rRNA and β-actin. The results of triplicate qPCR from three Reverse Transcription reactions are reported as a column plot of the mean ± s.e.m. of 47S rRNA/actin mRNA. (**d**) RNA isolated from cells treated as described in panel a, was analyzed by RT-qPCR with primers specific for RPS19, RPS6, RPL11 and β-actin. The results of triplicate RT-qPCR from three independent RNA preparations are reported as a column plot of the mean ± s.e.m. of RP mRNA/actin mRNA.

A number of publications have reported that interference with RP expression affects rRNA maturation^17^,^18^,^19^,^20^. We observed the same phenomenon in a K562 modified cell line (K562C), which is stably transduced with a lentiviral vector inducible for the expression of siRNA specific for RPS19 mRNA^21^. As shown in Fig. 1, doxycycline treatment of K562C induces alteration in the processing of pre-rRNA, indicated by accumulation of 21S pre-rRNA on a Northern blot probed with an oligonucleotide that anneals to the 5′ end of ITS1. In these experiments we noticed that the level of 47S precursor was also affected and decided to further address rRNA synthesis. To determine if RP depletion causes also a decrease of rRNA synthesis, we analyzed the level of the short-lived 47S pre-rRNA in a number of cell lines which had been manipulated to inhibit the synthesis of specific RPs: 1) K562C and TF-1C cells (similarly inducible for RPS19 depletion) treated with doxycycline (+dox); 2) HEK293 and 22RV1 cells transiently transfected with siRNA specific for RPS19; 3) K562 cells transiently transfected with siRNA specific for RPS19, RPS6 and RPL11. As a control, we used 1) untreated K562C and TF-1C cells (−dox), 2) K562S cells inducible for the expression of a scrambled siRNA treated with doxycycline (+dox); or 3) transient transfection of unrelated siRNA (siCNT). RNA levels were analyzed either by northern blot or by quantitative RT-PCR and the results are reported in Fig. 2. In all the cell lines, the depletion of RPS19 or the other RPs, verified by RT-qPCR, caused a reduction of 47S rRNA precursor to about 40–60% with respect to controls, that include doxycyclin-treated K562 cells (Fig. 2a,b). In addition, RPS19 overexpression after its siRNA-mediated depletion in HEK293 cells causes a recovery of 47S rRNA level (Fig. 2c), showing the effect is due to altered RPS19 levels.

### 47S rRNA stability and synthesis

**Figure 3:**
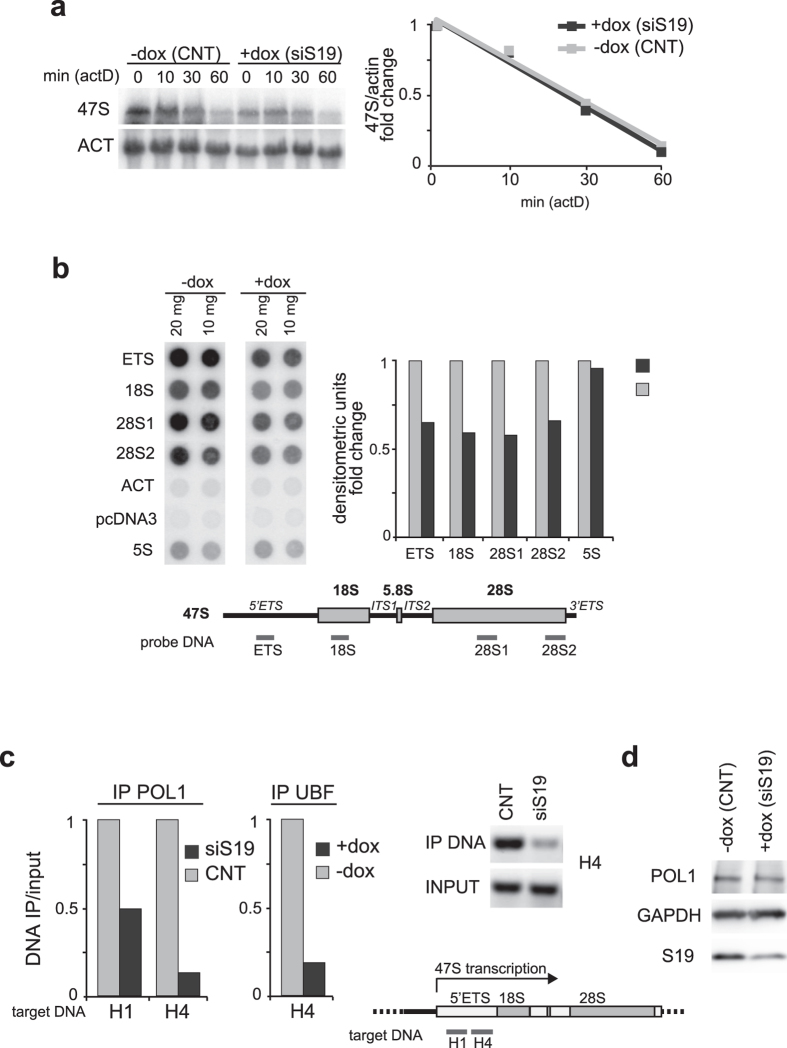
Analysis of rRNA stability and synthesis. (**a**) Control (−dox) or RPS19-depleted (+dox) K562C cells were treated with actinomycin D (20 ng/ml) for different times (0, 10′, 30′ and 60′) and total RNA was analyzed by Northern blot with probes specific for 5′ETS region of 47S rRNA and β-actin mRNA. Uncropped original images are shown in Supplementary information. Quantification of the signals is reported in the graph. (**b**) Nuclear run-on assay. Different amount of plasmid DNA (10 or 20 μg) including sequences of 5′ETS, 18S, two regions of 28S, β-actin mRNA, pCDNA3 and rRNA 5S were spotted on membrane and incubated with ^32^P-labeled RNA from untreated (−dox) or RPS19-depleted (+dox) K562C cells (left panel). The average of the two signals (10 and 20 μg) normalized for β-actin, is reported in the bar graph in the right panel. A schematic diagram of 47S pre-rRNA indicating the regions used in nuclear run-on assay is shown in the lower panel. (**c**) Chromatin cross-linking and immunoprecipitation assay (ChIP) was performed with Pol I-specific antibodies in HEK293 cells transfected for 72 h with control (CNT) or RPS19-specific siRNA (siS19), and with UBF-specific antibodies in K562C cells untreated (−dox) or incubated with doxycycline for 4 days (+dox). Associated DNA was analyzed by qPCR using primers specific for a sequence around the promoter (H1) or within the coding region (H4) of human rDNA. Right panel shows an ethidium bromide-stained agarose gel of input and Pol I-associated DNA (H4 region) amplified by PCR. The positions of H1 and H4 regions are shown in the lower panel. Uncropped original images are shown in Supplementary information. (**d**) Total extracts from K562C cells untreated (−dox) or incubated with doxycycline for 4 days (+dox) were analyzed by Western blot with the indicated antibodies. Uncropped original images are shown in Supplementary information.

To investigate whether the reduction of 47S rRNA was due to an increase of rRNA turnover, we treated the cells with a Pol I inhibitor while monitoring the kinetics of rRNA degradation both in control and RPS19-depleted cells. K562C cells were treated for different times (0, 10, 30 and 60 min) with low doses (20 ng/ml) of Actinomycin D (Act D), and the levels of rRNA species were analyzed by Northern blot. The results, reported in Fig. 3a, indicate that the rate of rRNA degradation did not increase in RPS19-deficient cells. This suggests that 47S decrease induced by RPS19 deficiency is not due to faster decay but likely reflects inhibition of transcription.

To address the hypothesis that RPS19 depletion inhibits the transcriptional activity of Pol I, we performed a nuclear run-on assay which, by measuring the amount of chromatin-bound (active) polymerase, allows one to evaluate the transcription initiation rate. The results show that Pol I activity in several different regions of rRNA genes is reduced after RPS19 depletion. No effect was observed for Pol III-dependent transcription of 5S rRNA genes, or for the Pol II-dependent transcription of the actin gene which serve as controls (Fig. 3b). Therefore, our results indicate that RPS19 depletion inhibits specifically the activity of Pol I.

To further study Pol I activity during ribosomal stress, we performed a chromatin immunoprecipitation assay (ChIP) in HEK293 cells after the depletion of RPS19 to analyze the binding of Pol I around the promoter (H1) and within the coding region (H4) of the major rRNA genes. In addition, we analyzed the binding of UBF within the H4 region in control and RPS19-depleted K562C cells. As shown in Fig. 3c, RPS19 deficiency causes a decrease in the binding of Pol I to both portions of rDNA and of UBF to H4. In fact, the presence of both H1 and/or H4 DNA segments in Pol I or UBF immunoprecipitate is reduced in RPS19-depleted cells (Fig. 3c). As an additional control we measured the level of Pol I by Western blot with specific antibodies of control and RPS19-depleted K562C cells. As shown in Fig. 3d, Pol I level is not affected by RPS19 depletion

### Pol I regulators affected by ribosomal stress

To address the mechanism underlying the inhibition of rRNA synthesis, we measured the level and the phosphorylation status of known regulators of Pol I activity. On the basis of preliminary analysis and published reports^22^,^23^,^24^, we considered the following proteins: 1) cyclin-dependent kinase 2 (CDK2), 2) AKT (protein kinase B), and 3) the AMP-activated protein kinase (AMPK).

CDK2 is an important component of the cell cycle machinery. We have previously reported that RPS19 depletion causes inhibition of cell cycle progression, a proliferation decline and an increase in the CDK inhibitor p27kip1 25. Pol I transcription is enhanced via phosphorylation of the basal transcription factor UBF by CDK2/cyclin complexes^24^. Thus, it can be hypothesized that in our experimental models after ribosomal stress, p27^kip1^ binds to and inhibits CDK2 activity leading to downregulation of rRNA synthesis.

The second regulatory protein analyzed is AKT, which is involved in signaling via both mammalian target of rapamycin complexes, mTORC1 and mTORC2. mTORC1 drives ribosome biogenesis^26^, by promoting activation of Pol I and Pol III and the synthesis of RPs. mTORC2 has been shown to interact with ribosomes, and knockdown of ribosomal proteins causes a decrease in mTORC2 signaling^27^. In addition, AKT has been reported to be necessary for Pol I–driven rDNA transcription^22^ and we have recently shown that it is involved in the response to ribosomal stress^28^.

Finally, a number of observations in our experimental models as well as published studies, are consistent with the activation of AMPK in response to ribosomal stress^23^,^29^. AMPK is activated under many cellular stress conditions such as hypoxia, nutrient deprivation or oxidative stress^30^. It has been shown that AMPK down-regulates rRNA synthesis under glucose restriction by phosphorylating the Pol I-associated transcription factor TIF-IA^31^. Therefore, this kinase was also a good candidate to be a component of the signaling activated by ribosomal stress.

**Figure 4:**
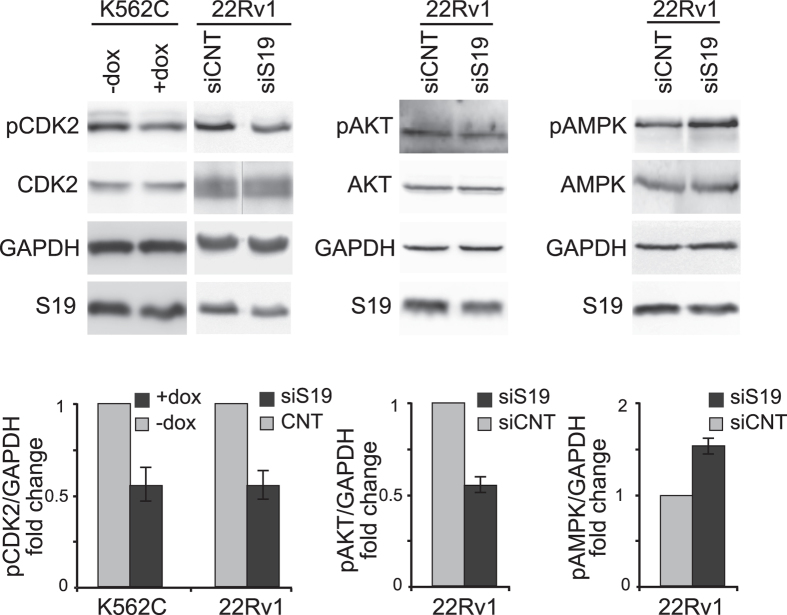
Protein analysis. Total extracts from K562C cells untreated (−dox) or treated with doxycycline for 4 days to induce RPS19 depletion (+dox) or from 22Rv1 cells transfected with control (siCNT) or RPS19-specific siRNA (siS19) were analyzed by Western blot with the indicated antibodies. Examples of Western from a single experiment are shown in the upper panels. Quantification of signals from three independent experiments is reported in lower panels as a column plot of the mean ± s.e.m. of the densitometry values normalized by GAPDH. The image of total CDK2 in 22Rv1 cells was obtained by cropping two lanes from a single blot. Uncropped original images are shown in Supplementary information.

To analyze the activity of Pol I regulators, K562C cells (only for CDK2) or 22Rv1 cells (for all three) were treated to induce RPS19 deficiency. Cell extracts were analyzed by western blot with antibodies specific for CDK2 total or phosphorylated on Thr160, AKT total or phosphorylated on Ser473, AMPK total or phosphorylated on Thr172 plus ‘loading’ controls (RPS19 and GAPDH). The results, reported in Fig. 4, show that RPS19 depletion causes a reduction of the active forms of CDK2 and AKT (both positive regulators of Pol I) and an increase in the phosphorylation of Thr172, a key activating site in AMPK (an inhibitor of Pol I). Thus, all three kinases could potentially mediate the downregulation of Pol I activity caused by ribosomal stress.

### 47S rRNA levels in DBA patients

**Figure 5:**
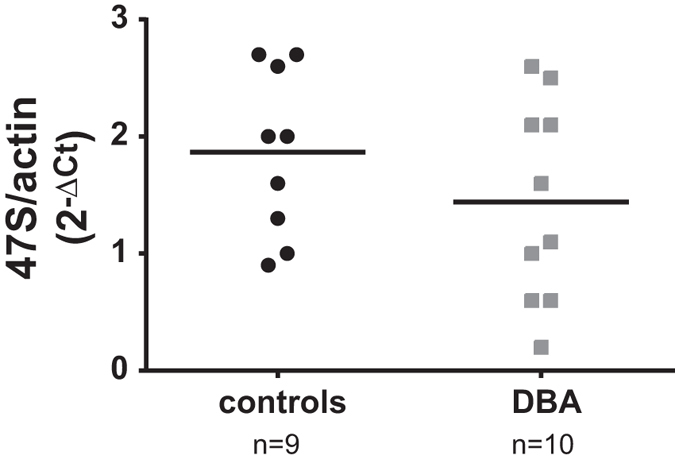
47S rRNA level in cells from DBA patients. Total RNA from mononuclear cells of 9 controls or 10 DBA patients harboring different mutations (indicated in Table 1) were analyzed by RT-qPCR with primers specific for 47S and β-actin. The results of triplicate RT-qPCR analyses from each sample are reported as a dot plot of 47S rRNA level calculated with the 2^−ΔCt^ method. The orizontal bars indicate the means of the two groups of values.

Alteration of rRNA processing has been observed in cultured cells after depletion of RPs and confirmed to occur in cells from DBA patients^18^,^19^,^32^. It has been proposed that this finding could even be exploited as a diagnostic tool^33^. For this reason, we decided to investigate if the decrease in rRNA synthesis caused by RP depletion could be detected in samples from DBA patients. To this end, RNA was purified from blood samples of 9 control donors (4 males, 5 females; age from 9 to 55) and 10 DBA patients (6 males, 4 females; age from 5 to 40). Ribosomal RNA level was analyzed by quantitative RT-PCR with primer specific for the first part of the ETS region in the 47S rRNA precursor and for β-actin mRNA as a control. The results, reported in Fig. 5 and Table 1, indicate that, on average, the level of the 47S pre-rRNA species is lower in DBA patients compared to controls. However the large variability in 47S levels, both in the patients and in the controls does not allow this analysis to be statistically significant and to be used as diagnostic tool. It could be that increasing the number of samples and/or the stratification of the patients into different subsets will render the analysis more meaningful.

## Discussion

Considering the complexity and importance of ribosome biogenesis, it seems probable that a number of mechanisms actively coordinate the production of the different components required to make functional ribosomes. Identification of such regulatory circuits may reveal important details of the pathological mechanisms of ribosomopathies. One of these controls has been shown to operate at the level of rRNA processing. In fact, the deficiency in any RP causes a block in rRNA maturation with the accumulation of processing intermediates^17^,^34^. Here we report an additional mechanism that works at the level of rRNA synthesis by inhibiting the activity of Pol I in response to RP deficiency, i.e., at a very early stage in ribosome production.

The effects of RPS19 deficiency were analyzed in human cultured cells manipulated to express specific siRNAs. These included: the erythroleukemia cell lines K562C and TF-1C inducible for the expression of siRNA against RPS19 mRNA; the prostate cancer cell line 22Rv1 and the embryonic kidney cell line HEK293 both transiently transfected with siRNA for RPS19. In addition, K562 cells were transiently transfected with siRNA specific for RPS19, RPS6 or RPL11. In all cases, we observed that depletion of RPs caused a decrease in the level of 47S rRNA, analyzed by Northern blot and/or RT-qPCR. Further analysis indicated that the lower level of 47S rRNA was due to a reduction in the activity of Pol I. In fact, treatment of K562C cells with the transcription inhibitor actinomycin D showed that the stability of 47S pre-rRNA was not affected by RP depletion. Moreover, run-on assays and ChIP experiments showed that RPS19 deficiency caused a reduction of Pol I activity on rDNA and a reduction of the binding of UBF, which is a regulator of Pol I activity, on rRNA^5^. We conclude that we have identified a novel regulatory circuit which coordinates the synthesis of rRNA with the availability of RPs.

It has been shown that most of RPs of the small subunit associate with pre-rRNA and at least some of them are required for processing^18^,^20^,^35^. We think that it is unlikely that RP interaction with rRNA could directly affect Pol I activity. In addition, proteomic studies indicated an interaction between RPS19 and UBF^36^ that could play a regulatory role. However, depletion of RPS6 or RPL11 show an inhibitory effect on rRNA synthesis similar to RPS19 and it is improbable that multiple RPs regulate Pol I activity by direct interaction. As an alternative, we hypothesize that a signaling pathway, activated by defective RP synthesis, targets the transcriptional apparatus for rDNA genes. Through immunoblot analysis, we identified several known regulators of Pol I that are affected by ribosomal stress. Firstly, in RPS19-deficient cells CDK2 is hypophosphorylated on the activatory site Thr160. It has been demonstrated that different Pol I-associated transcription factors are positively regulated by this kinase such as TIF-IA and UBF^5^,^37^. In particular, it is known that after G1 progression, phosphorylation by CDK2 is required for UBF activity and represents a powerful means for modulating the assembly of the transcription initiation complex^24^. The reduction of CDK2 activity caused by RPS19 depletion may therefore inhibit the recruitment of UBF.

A second molecule which may be involved is AKT. We found that RPS19 depletion caused a decrease in AKT phosphorylation on Ser473. AKT signaling activates mTORC1, which drives multiple steps in ribosome biogenesis including rRNA synthesis^26^. AKT is required for Pol I driven rRNA transcription at multiple levels, including loading of Pol I onto the rDNA (transcription initiation and promoter escape), and subsequent steps, such as transcription elongation and rRNA processing^22^. Since in our experimental setup ribosomal stress induced a decrease of AKT phosphorylation, the deficiency of AKT activity may contribute to the suppression of Pol I activity. Ser473 is phosphorylated by mTORC2, which is maintained in an active state through its interaction with ribosomes^27^; thus, depletion of an RP may compromise the activation state of mTORC2 leading to reduced phosphorylation of AKT at Ser473, which is involved in its activation.

A third established regulator of Pol I is AMPK which is also activated by ribosomal stress. It has been proposed that RP depletion could activate AMPK through the alteration of nucleotide metabolism caused by the degradation of excess rRNA^23^. It is known that, when activated, AMPK down-regulates rRNA synthesis by phosphorylating the RNA polymerase I (Pol I)-associated transcription factor TIF-IA. Phosphorylation by AMPK impairs the interaction of TIF-IA with the TBP-containing promoter selectivity factor SL1, thereby precluding the assembly of functional transcription initiation complex^31^. Therefore this kinase could also contribute to the reduction of Pol I activity during ribosomal stress. At this stage, we do not know whether one or more of these factors is responsible of the downregulation of Pol I activity during ribosomal stress. It is also unclear if the observed Pol I inhibition is a consequence or one of causes of nucleolar alteration induced by RP depletion^38^,^39^. Although RP depletion is not always associated to nucleolar disruption^40^. Further studies are needed to address this issues.

Finally, we tested if the inhibition of Pol I activity in response to ribosomal stress could be observed in the physiological context of blood cells from DBA patients. For this analysis, blood samples were collected from 9 control donors and 10 DBA patients and RNA was analyzed by RT-qPCR. We found that, on average, the 47S rRNA level was lower in RNA samples from DBA patients compared to controls. However, the variability of values does not allow an unambigous classification of the samples. Additional analysis may lead to the identification of a subgroup of patients with more homogeneous values.

## Methods

### Cell culture and transient transfection

K562C and TF-1C (human erythroleukemia) cells were maintained in RPMI 1640 medium. HEK293 (human embryonic kidney) and 22RV1 (human prostate carcinoma epithelial) cells were maintained in Dulbecco’s modified Eagle Medium (DMEM) supplemented with 10% fetal bovine serum, and antibiotics (100 U/ml penicillin and 100 μg/ml streptomycin) and were incubated at 37 °C in a humidified atmosphere with 5% CO_2_. TF-1C medium was also supplemented with 5 ng/ml Granulocyte- Macrophage Colony-Stimulating Factor. TF-1C and K562C cells, expressing inducible siRNA targeting RPS19 mRNA, were prepared in Karlsson’s laboratory^21^. K562, HEK293 and 22RV1 (3 × 10^6^) were transiently transfected with 100 nM siRNA and 15 μl of Interferin transfection reagent (Polyplus transfection) according to the manufacturer’s protocol. After 48 hours they were re-transfected and 48 hours later were harvested and analyzed by RT-qPCR. The RNAi target sequences were: sense, 5′-GAGAUCUGGACAGAAUCGC-3′ for RPS19 and sense 5′-GACACGCGACUUGUACCAC-3′ for control siRNA (siSCR). In the case of RPS19 overexpression, HEK293 cells, after double siRNA treatment, were transfected with a RPS19-expressing plasmid (6 μg) 24 hours before harvest. Actinomycin treatment of cells was performed using 20 ng/ml for the indicated times.

### RNA preparation and northern blot analysis

Total RNA was extracted from cell lines by the proteinase K method^41^. For Northern analysis, RNA was fractionated on formaldehyde-agarose gels and transferred to GeneScreen Plus membrane (PerkinElmer Life Sciences), as previously described^42^. Probe for β-actin was prepared by the random primer technique using DNA fragments isolated from plasmids containing PCR amplified cDNA sequences. Primers for amplification were designed according to sequences present in the NCBI Data Bank. For rRNA probes, an oligonucleotide (GTGAGCACGACGTCACCACATCGATCGAAGATC) complementary to human rRNA sequence (NR_046235) from position 431 (5′ETS) and an oligonucleotide (CCTCGCCCTCCGGGCTCCGTTAATTGATC) complementary to human rRNA sequence (NR_046235) from position 5520 (ITS1), were radiolabeled with [γ−^32^P]-ATP^41^. Quantitation of Northern blot filters was performed with a PhosphorImager (GE Healthcare).

For RNA analysis of DBA patients and controls, a written informed consent was signed by patients and controls or their parents or tutors. Blood was collected into PAXgene Blood RNA Tubes (PreAnalytiX) and RNA was purified using the PAXgene Blood RNA kit (PreAnalytiX) following manufacturer’s instructions.

### Western blot analysis

Cells were lysed in lysis buffer containing 350 mM NaCl, 1 mM MgCl2, 50 mM Tris–HCl (pH 7.5), 0.5 mM EDTA, 0.1 mM EGTA, 1% NP-40, aprotinin 1 mg/ml, phenylmethylsulfonyl fluoride 100 mg/ml and 1% [vol/vol] phosphatase inhibitor cocktail II and III from sigma). Protein concentration was measured by Bio-Rad Bradford reagent. Protein sample were prepared by addition of Laemli Sample buffer and resolved on 8–10% SDS-PAGE (Sodium dodecyl sulfate–polyacrylamide gel), transferred onto nitrocellulose Protran membrane (Schleicher and Schuell, Italy), and incubated with the following primary antibodies and antisera: mouse monoclonal antibody specific for RPS19^36^, mouse monoclonal anti-GAPDH (Millipore), mouse monoclonal anti-Pol I (Santa Cruz Biotechnology, USA), mouse monoclonal anti-total CDK2 (Santa Cruz Biotechnology, USA), rabbit polyclonal anti-phospho CDK2 Thr160 (Cell Signaling), rabbit polyclonal anti-phospho AKT Ser473 (Cell Signaling Technologies, USA), rabbit polyclonal anti-total AKT (Cell Signaling Technologies, USA), rabbit monoclonal anti-phospho AMPK Thr172 (Cell Signaling Technologies, USA), mouse monoclonal anti-total AMPK (Santa Cruz Biotechnology, USA). Primary antibodies were revealed using horseradish peroxidase-conjugated anti-rabbit or anti-mouse Ab (Jackson Immunoresearch) and the ECL Clarity Western substrate detection (BIO-RAD). Quantification analyses were performed by LAS3000 Image System (Fuji) and ImageQuant software (GE Healthcare).

### Chromatin cross-linking and immunoprecipitation (ChIP)

HEK293 cells were treated for 72 h with RPS19-specific siRNA and K562C were treated for 4 days with doxycycline. Cross-linking was performed by incubating cells with 1% formaldehyde for 10 min at 37 °C. The cells were lysed in 350 μl of lysis buffer (1% SDS, 10 mM EDTA, 50 mM Tris-HCl, pH 8.1) and sonicated seven times for 20 s. Extracts were immunoprecipitated with antibodies specific for Pol I (Santa Cruz) and UBF (Santa Cruz) and DNA isolated from precipitates was used for qPCR analysis with primers specific for rDNA region H1 (5′-GGCGGTTTGAGTGAGACGAGA-3′; 5′-ACGTGCGCTCACCGAGAGCAG-3′) and H4 (5′-CGACGACCCATTCGAACGTCT-3′; 5′-CTCTCCGGAATCGAACCCTGA-3′). Input DNA was used for normalization. The samples were analysed with SYBR Green dye (Primer Design mix) on an ABI StepOnePlus quantitative PCR instrument (Applied Biosystems).

### RT-qPCR analysis

Total RNA (2 μg) was reverse transcribed to cDNA using MMLV enzyme (Promega) with random primers following the manufacturer’s protocol. Quantitative PCR on cDNA from cell lines was performed using specific primers for human 47S (5′-GCTGACACGCTGTCCTCTG-3′; 5′-ACGCGCGAGAGAACAGCAG-3′) and β-actin (5′-ACCACCATGTACCCTGGCATT-3′; 5′-CCACACGGAGTACTTGCGCTCA-3′). The samples were analysed in triplicate with KAPA SYBR FAST (KAPABIOSYSTEMS) on an Roche Applied Science (LC480) quantitative PCR instrument. The comparative Ct method (2^−ΔCt^) was used to measure amplification of rRNA 47S versus β-actin.

Quantitative PCR on cDNA from DBA patients was carried out using a StepOne real-Time PCR detection system (Applied Biosystems). Taqman 2X Universal Master Mix II, no UNG (Applied Biosystems) was used according to the manufacturer’s instructions. To detect 47S rRNA precursor, the following oligos were used: forward,+132/+149 CCTGCTGTCTCTCGCGC; reverse+198/+181, GGTCAGAGACCCGGACCC; Taqman,+155/174 AGCGTCCCGACTCCCGGTGC. The Applied Biosystems Human β-actin Endogenus Control (FAMTM/MGB Probe, Non-Primer Limited) was used as an endogenous control.

### Nuclear run-on

Approximately 10^8^ K562C cells were lysed in hypotonic buffer (10 mM Tris pH 7.9, 10 mM NaCl, 5 mM MgCl_2_) containing 0.1% NP40 and nuclei were purified through a 0.6 M sucrose cushion. Nuclei were resuspended in 50 mM Tris pH 8.5, 10 mM NaCl, 5 mM MgCl2, 40% glycerol, 1 mM DTT and then frozen. About 2 × 10^7^ nuclei were incubated for 5 min at 25 °C in a buffer containing 150 mM KCl, 1 mM NTP, 0.05 mCi α-32P GTP. Labeled RNA was extracted using proteinase K method and resuspended in hybridization buffer (GeneScreen Plus). Plasmids containing different regions of rDNA were prepared by inserting PCR products into pGemTEasy vector. Cloned sequences, reported by name, start and stop nucleotide position detectable on human ribosomal DNA complete repeating unit (GI: 555853) and length in base pairs, are the following: 5′-ETS (from 1286 to 1920, 635 bp); 18S (from 4101 to 4721, 621 bp); 28S1 (from 9561 to 10320; 760 bp); 28S2 (from 12207 to 12930; 724 bp). Plasmid DNAs were immobilized on a GeneScreen Plus membrane using a dot-blot apparatus. Hybridization was carried out as indicated in GeneScreen Plus manual.

## Additional Information

**How to cite this article**: Juli, G. *et al*. Depletion of ribosomal protein S19 causes a reduction of rRNA synthesis. *Sci. Rep.* **6**, 35026; doi: 10.1038/srep35026 (2016).

## Supplementary Material

Supplementary Information

## Figures and Tables

**Table 1:** 47S rRNA level in RNA samples from DBA patients.

RNA sample	Mutation	47S/ACTIN
1	RPL11	2.5
2	unknown	1.1
3	RPS19	1.6
4	RPS19	1
5	unknown	2.1
6	unknown	0.6
7	unknown	0.2
8	RPL11	2.1
9	RPL5	0.6
10	RPL5	2.6

RNA was analyzed by RT-qPCR with primers specific for 47S and β-actin in triplicate. The values were calculated with the 2^−ΔCt^ method.

## References

[b1] Goodfellow S. J. & Zomerdijk J. C. Basic mechanisms in RNA polymerase I transcription of the ribosomal RNA genes. Sub-cellular biochemistry 61, 211–236 (2013). 23150253 10.1007/978-94-007-4525-4_10 PMC3855190

[b2] Caldarola S., Amaldi F., Proud C. G. & Loreni F. Translational regulation of terminal oligopyrimidine mRNAs induced by serum and amino acids involves distinct signaling events. J Biol Chem 279, 13522–13531 (2004). 14726531 10.1074/jbc.M310574200

[b3] Caldarola S., De Stefano M. C., Amaldi F. & Loreni F. Synthesis and function of ribosomal proteins–fading models and new perspectives. FEBS J 276, 3199–3210 (2009). 19438715 10.1111/j.1742-4658.2009.07036.x

[b4] Henras A. K. . The post-transcriptional steps of eukaryotic ribosome biogenesis. Cell Mol Life Sci 65, 2334–2359 (2008). 18408888 10.1007/s00018-008-8027-0 PMC11131730

[b5] Drygin D., Rice W. G. & Grummt I. The RNA Polymerase I Transcription Machinery: An Emerging Target for the Treatment of Cancer. Annu. Rev. Pharmacol. Toxicol. 50, 131–156 (2010). 20055700 10.1146/annurev.pharmtox.010909.105844

[b6] Rouquette J., Choesmel V. & Gleizes P. E. Nuclear export and cytoplasmic processing of precursors to the 40S ribosomal subunits in mammalian cells. EMBO J 24, 2862–2872 (2005). 16037817 10.1038/sj.emboj.7600752 PMC1187937

[b7] Moss T. & Stefanovsky V. Y. At the center of eukaryotic life. Cell 109, 545–548 (2002). 12062097 10.1016/s0092-8674(02)00761-4

[b8] Ruggero D. & Pandolfi P. P. Does the ribosome translate cancer? Nat Rev Cancer 3, 179–192 (2003). 12612653 10.1038/nrc1015

[b9] Russell J. & Zomerdijk J. C. RNA-polymerase-I-directed rDNA transcription, life and works. Trends Biochem Sci 30, 87–96 (2005). 15691654 10.1016/j.tibs.2004.12.008 PMC3858833

[b10] Grummt I. Life on a planet of its own: regulation of RNA polymerase I transcription in the nucleolus. Genes Dev 17, 1691–1702 (2003). 12865296 10.1101/gad.1098503R

[b11] Grummt I. & Voit R. Linking rDNA transcription to the cellular energy supply. Cell Cycle 9, 225–226 (2010). 20023389 10.4161/cc.9.2.10614

[b12] Moss T. At the crossroads of growth control; making ribosomal RNA. Curr Opin Genet Dev 14, 210–217 (2004). 15196469 10.1016/j.gde.2004.02.005

[b13] Danilova N. & Gazda H. T. Ribosomopathies: how a common root can cause a tree of pathologies. Disease models & mechanisms 8, 1013–1026 (2015). 26398160 10.1242/dmm.020529 PMC4582105

[b14] Loreni F., Mancino M. & Biffo S. Translation factors and ribosomal proteins control tumor onset and progression: how? Oncogene 33, 2145–2156 (2014). 23644661 10.1038/onc.2013.153

[b15] Dianzani I. & Loreni F. Diamond-Blackfan anemia: a ribosomal puzzle. Haematologica 93, 1601–1604 (2008). 18978295 10.3324/haematol.2008.000513

[b16] Ellis S. R. Nucleolar stress in Diamond Blackfan anemia pathophysiology. Biochim Biophys Acta 1842, 765–768 (2014). 24412987 10.1016/j.bbadis.2013.12.013

[b17] Choesmel V. . Impaired ribosome biogenesis in Diamond-Blackfan anemia. Blood 109, 1275–1283 (2007). 17053056 10.1182/blood-2006-07-038372 PMC1785132

[b18] Idol R. A. . Cells depleted for RPS19, a protein associated with Diamond Blackfan Anemia, show defects in 18S ribosomal RNA synthesis and small ribosomal subunit production. Blood Cells Mol Dis 39, 35–43 (2007). 17376718 10.1016/j.bcmd.2007.02.001

[b19] Flygare J. . Human RPS19, the gene mutated in Diamond-Blackfan anemia, encodes a ribosomal protein required for the maturation of 40S ribosomal subunits. Blood 109, 980–986 (2007). 16990592 10.1182/blood-2006-07-038232 PMC1785147

[b20] O’Donohue M. F., Choesmel V., Faubladier M., Fichant G. & Gleizes P. E. Functional dichotomy of ribosomal proteins during the synthesis of mammalian 40S ribosomal subunits. J Cell Biol 190, 853–866 (2010). 20819938 10.1083/jcb.201005117 PMC2935573

[b21] Miyake K. . Development of cellular models for ribosomal protein S19 (RPS19)-deficient diamond-blackfan anemia using inducible expression of siRNA against RPS19. Mol Ther 11, 627–637 (2005). 15771965 10.1016/j.ymthe.2004.12.001

[b22] Chan J. C. . AKT promotes rRNA synthesis and cooperates with c-MYC to stimulate ribosome biogenesis in cancer. Sci Signal 4, ra56 (2011). 21878679 10.1126/scisignal.2001754

[b23] Danilova N. . The role of the DNA damage response in zebrafish and cellular models of Diamond Blackfan anemia. Disease models & mechanisms 7, 895–905 (2014). 24812435 10.1242/dmm.015495 PMC4073278

[b24] Voit R. & Grummt I. Phosphorylation of UBF at serine 388 is required for interaction with RNA polymerase I and activation of rDNA transcription. Proc Natl Acad Sci USA 98, 13631–13636 (2001). 11698641 10.1073/pnas.231071698 PMC61092

[b25] Iadevaia V. . PIM1 kinase is destabilized by ribosomal stress causing inhibition of cell cycle progression. Oncogene 29, 5490–5499 (2010). 20639905 10.1038/onc.2010.279

[b26] Iadevaia V., Huo Y., Zhang Z., Foster L. J. & Proud C. G. Roles of the mammalian target of rapamycin, mTOR, in controlling ribosome biogenesis and protein synthesis. Biochem Soc Trans 40, 168–172 (2012). 22260684 10.1042/BST20110682

[b27] Zinzalla V., Stracka D., Oppliger W. & Hall M. N. Activation of mTORC2 by association with the ribosome. Cell 144, 757–768 (2011). 21376236 10.1016/j.cell.2011.02.014

[b28] Sagar V. . PIM1 destabilization activates a p53-dependent response to ribosomal stress in cancer cells. Oncotarget (2016). 10.18632/oncotarget.8070 PMC5029667 26993775

[b29] Gismondi A. . Ribosomal stress activates eEF2K-eEF2 pathway causing translation elongation inhibition and recruitment of terminal oligopyrimidine (TOP) mRNAs on polysomes. Nucleic Acids Res 42, 12668–12680 (2014). 25332393 10.1093/nar/gku996 PMC4227798

[b30] Mihaylova M. M. & Shaw R. J. The AMPK signalling pathway coordinates cell growth, autophagy and metabolism. Nat Cell Biol 13, 1016–1023 (2011). 21892142 10.1038/ncb2329 PMC3249400

[b31] Hoppe S. . AMP-activated protein kinase adapts rRNA synthesis to cellular energy supply. Proc Natl Acad Sci USA 106, 17781–17786 (2009). 19815529 10.1073/pnas.0909873106 PMC2764937

[b32] Choesmel V. . Mutation of ribosomal protein RPS24 in Diamond-Blackfan anemia results in a ribosome biogenesis disorder. Hum Mol Genet 17, 1253–1263 (2008). 18230666 10.1093/hmg/ddn015

[b33] Quarello P. . Ribosomal RNA analysis in the diagnosis of Diamond-Blackfan Anaemia. Br J Haematol 172, 782–785 (2016). 26763766 10.1111/bjh.13880

[b34] Robledo S. . The role of human ribosomal proteins in the maturation of rRNA and ribosome production. RNA 14, 1918–1929 (2008). 18697920 10.1261/rna.1132008 PMC2525958

[b35] Ferreira-Cerca S. . Analysis of the *in vivo* assembly pathway of eukaryotic 40S ribosomal proteins. Mol Cell 28, 446–457 (2007). 17996708 10.1016/j.molcel.2007.09.029

[b36] Orru S. . Analysis of the ribosomal protein S19 interactome. Mol Cell Proteomics 6, 382–393 (2007). 17151020 10.1074/mcp.M600156-MCP200

[b37] Hein N., Hannan K. M., George A. J., Sanij E. & Hannan R. D. The nucleolus: an emerging target for cancer therapy. Trends Mol Med 19, 643–654 (2013). 23953479 10.1016/j.molmed.2013.07.005

[b38] Bursac S., Brdovcak M. C., Donati G. & Volarevic S. Activation of the tumor suppressor p53 upon impairment of ribosome biogenesis. Biochim Biophys Acta 1842, 817–830 (2014). 24514102 10.1016/j.bbadis.2013.08.014

[b39] Deisenroth C. & Zhang Y. Ribosome biogenesis surveillance: probing the ribosomal protein-Mdm2-p53 pathway. Oncogene 29, 4253–4260 (2010). 20498634 10.1038/onc.2010.189

[b40] Fumagalli S. . Absence of nucleolar disruption after impairment of 40S ribosome biogenesis reveals an rpL11-translation-dependent mechanism of p53 induction. Nat Cell Biol 11, 501–508 (2009). 19287375 10.1038/ncb1858 PMC4810440

[b41] Sambrook J., Fritsch E. F. & Maniatis T. Molecular Cloning: A Laboratory Manual. (Cold Spring Harbor Laboratory Press, 1989).

[b42] Iadevaia V., Caldarola S., Tino E., Amaldi F. & Loreni F. All translation elongation factors and the e, f, and h subunits of translation initiation factor 3 are encoded by 5′-terminal oligopyrimidine (TOP) mRNAs. RNA 14, 1730–1736 (2008). 18658124 10.1261/rna.1037108 PMC2525946

