# Supplementary material for: Depletion of ribosomal protein S19 causes a reduction of rRNA synthesis
Source: Sci Rep. 2016 Oct 13;6:35026. doi: 10.1038/srep35026 (PMC5062126; doi:10.1038/srep35026)

Depletion of ribosomal protein S19 causes a reduction of rRNA synthesis

Giada Juli[1], Angelo Gismondi[1], Valentina Monteleone[1], Sara Caldarola[1], Valentina Iadevaia[2], Anna Aspesi[3], Irma Dianzani[3], Christopher G. Proud[2] and Fabrizio Loreni[1]*

1) Department of Biology, University of Rome Tor Vergata, Roma, Italy

2) Centre for Biological Sciences, University of Southampton, Southampton, UK

3) Department of Health Sciences, Università del Piemonte Orientale, Novara, Italy

Supplementary information:

Original images of gels, Northern blots and Western blots. The parts of the images reported in the figures are indicated by red rectangles.

Figure 2b - Northern blot

47S

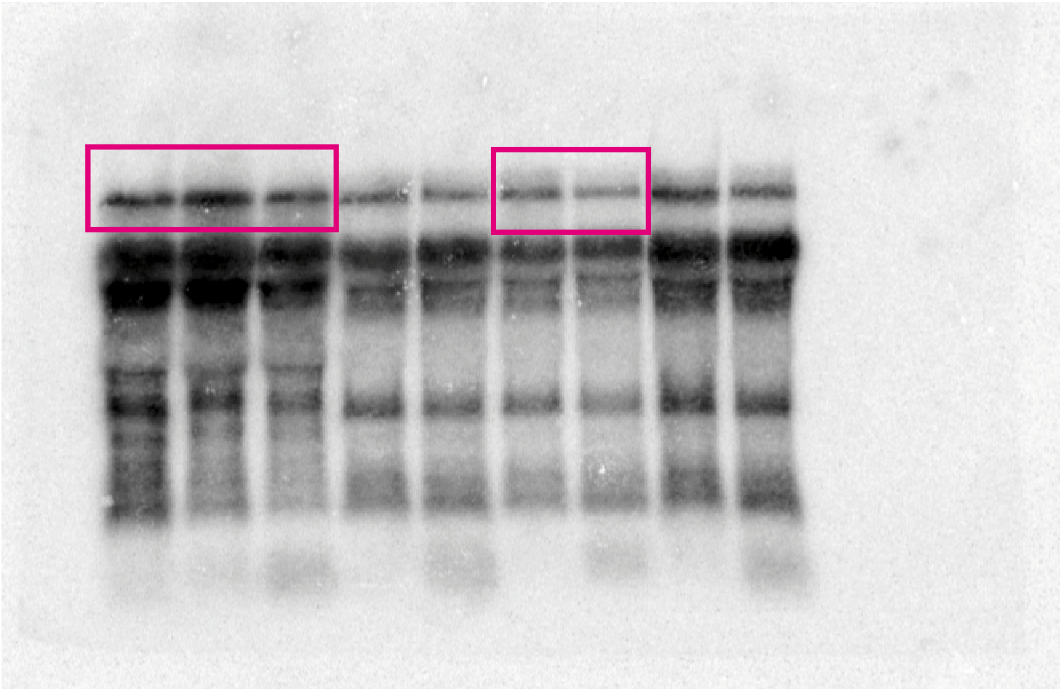

ACTIN

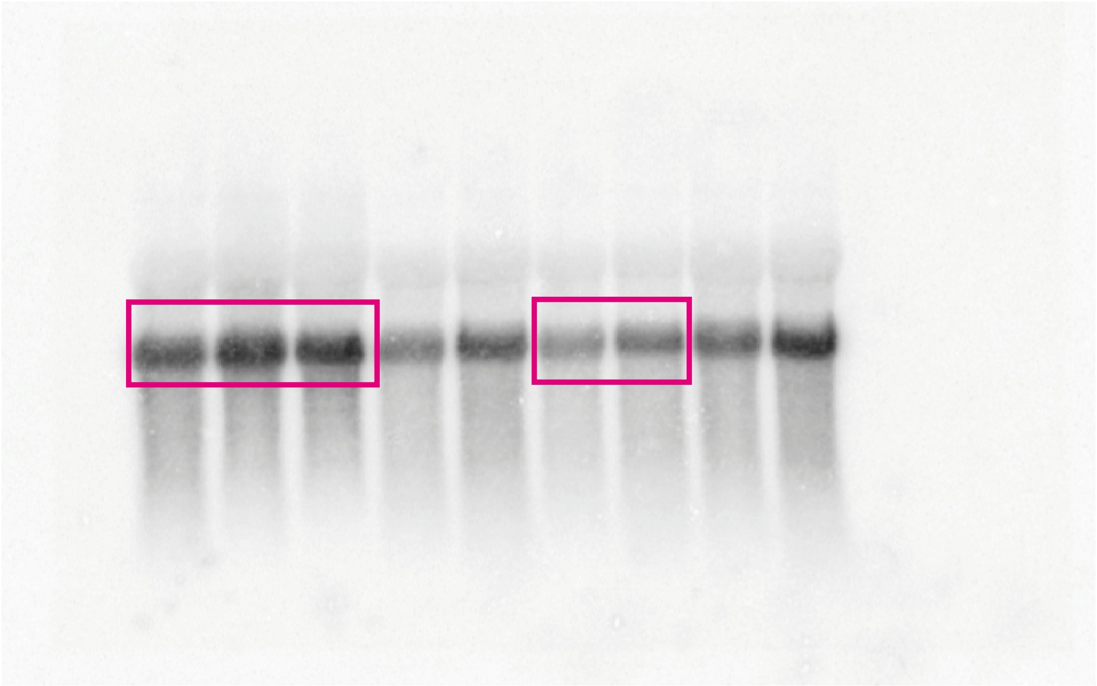

Figure 3a - Northern blot

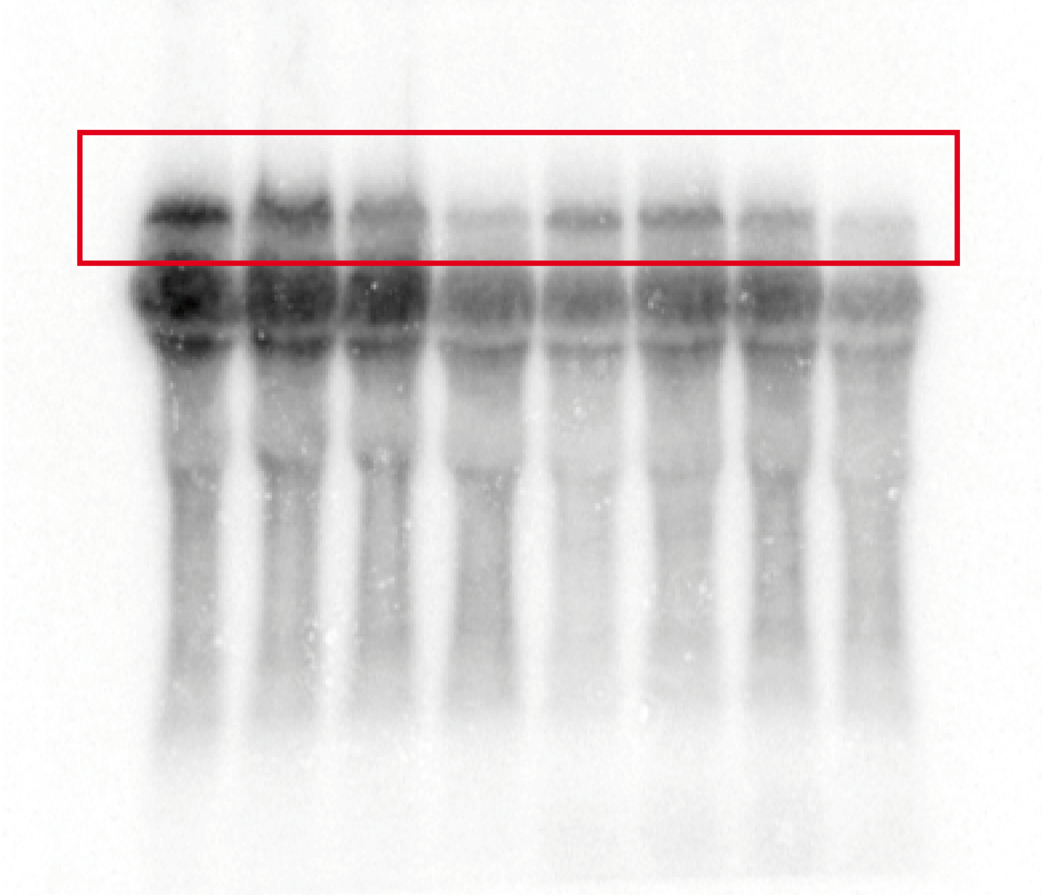

47S

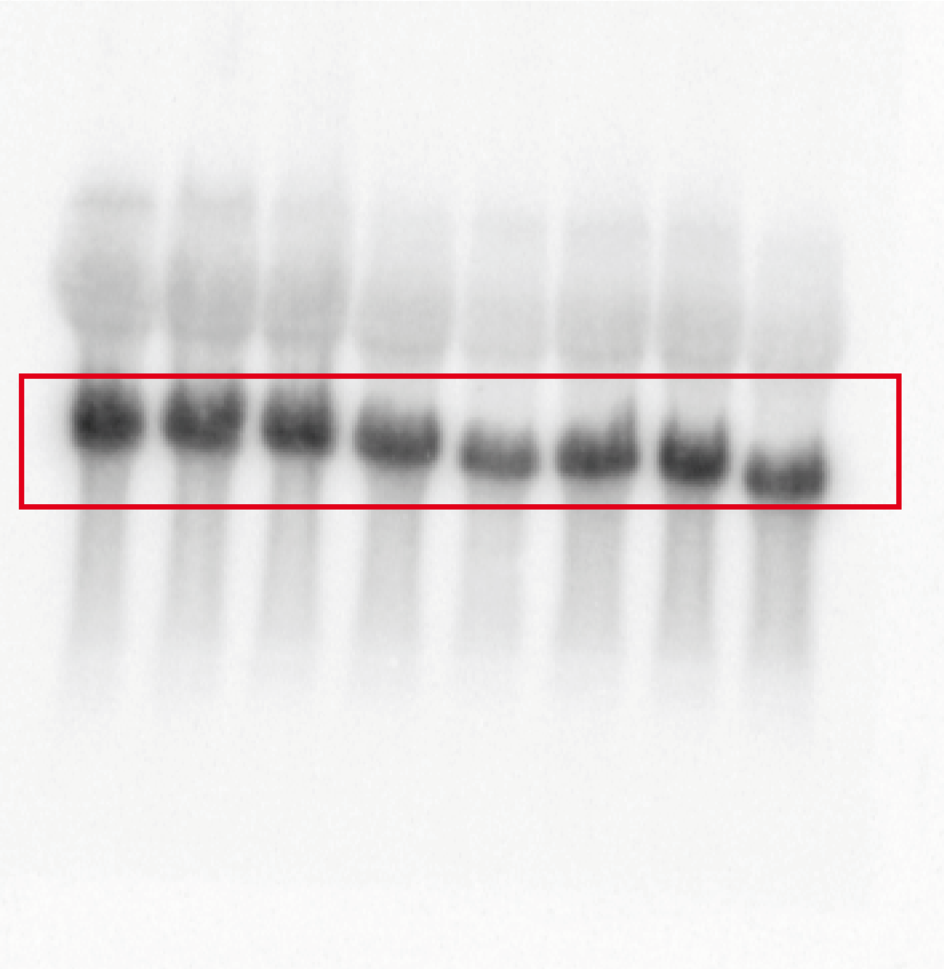

ACTIN

Figure 3c -Agarose gel

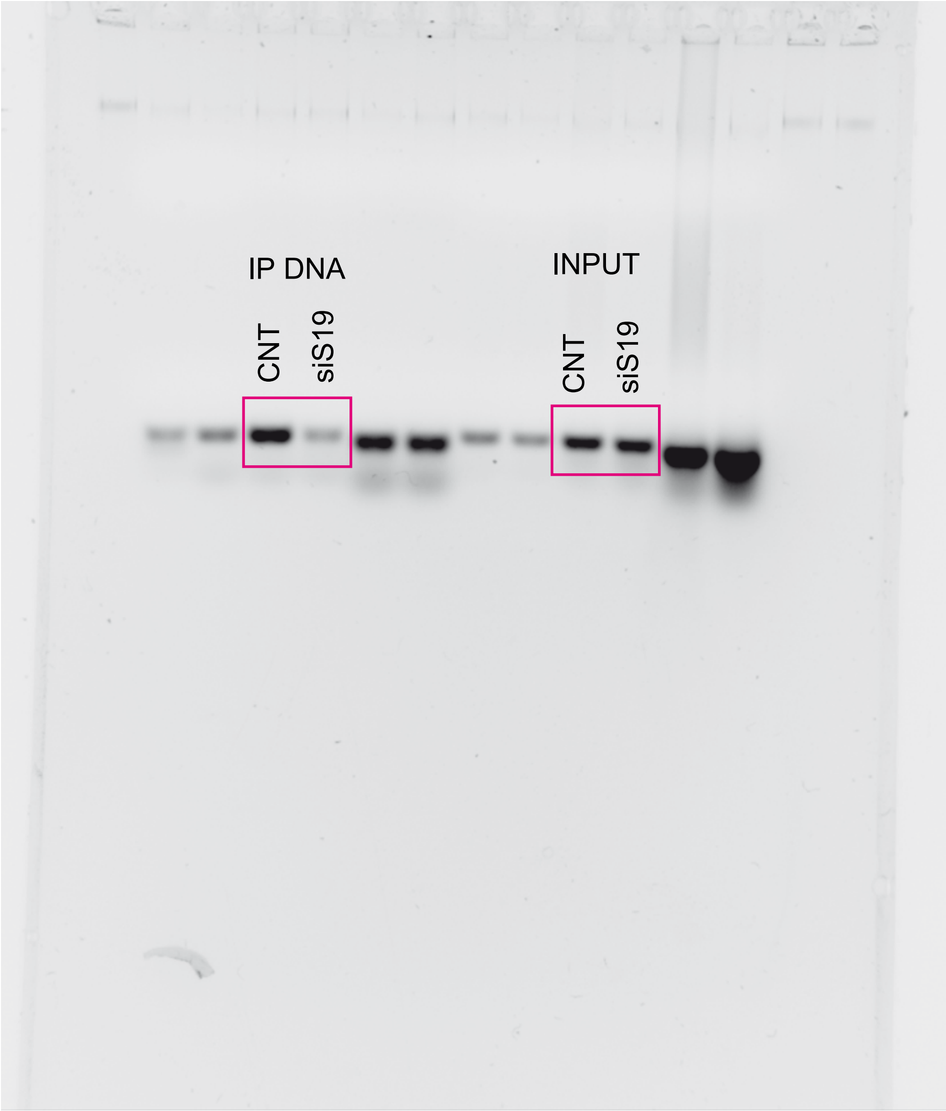

Figure 3d -Western blots

POL 1

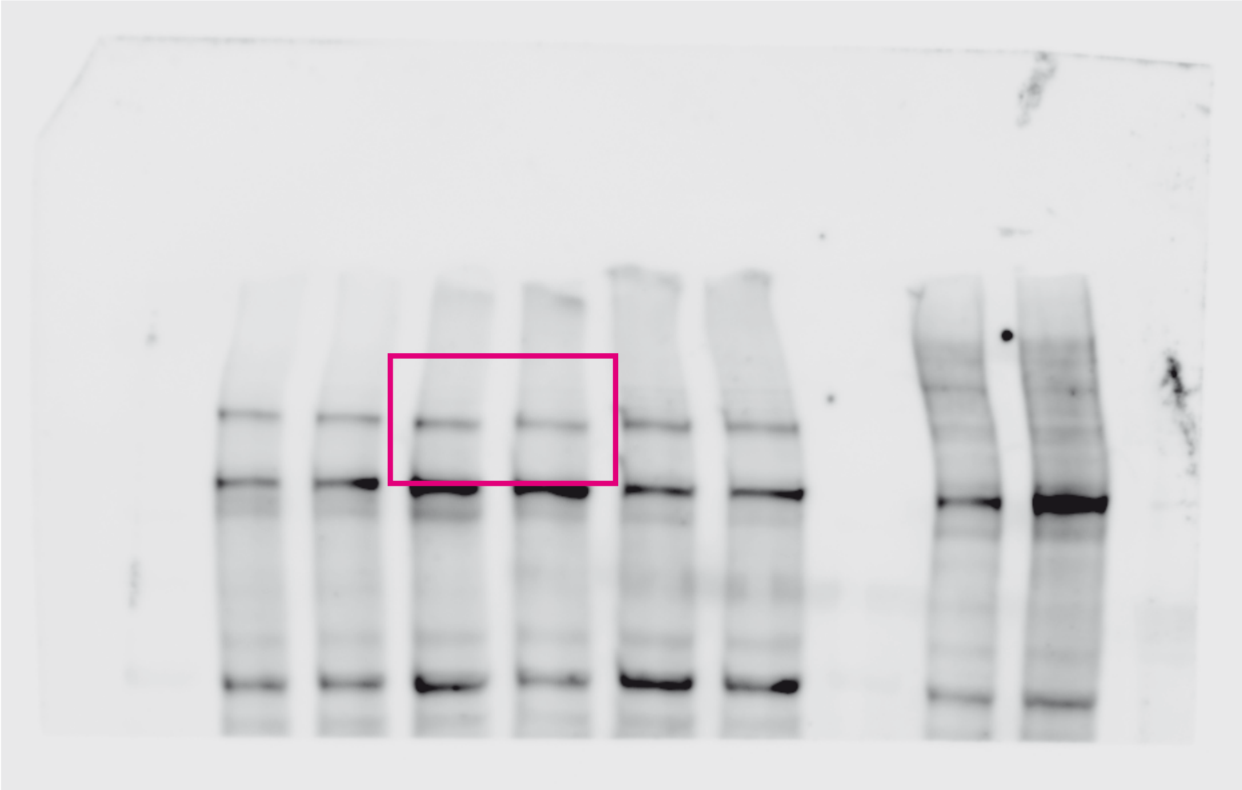

GAPDH

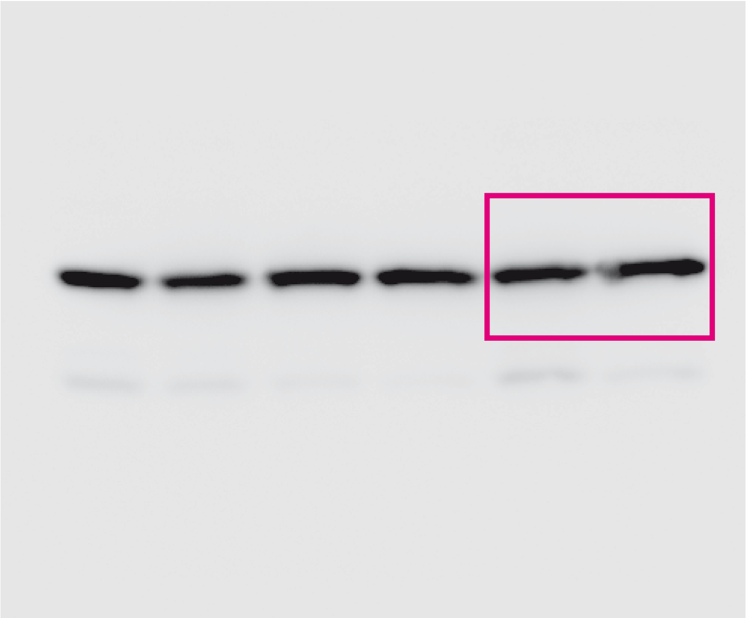

RPS19

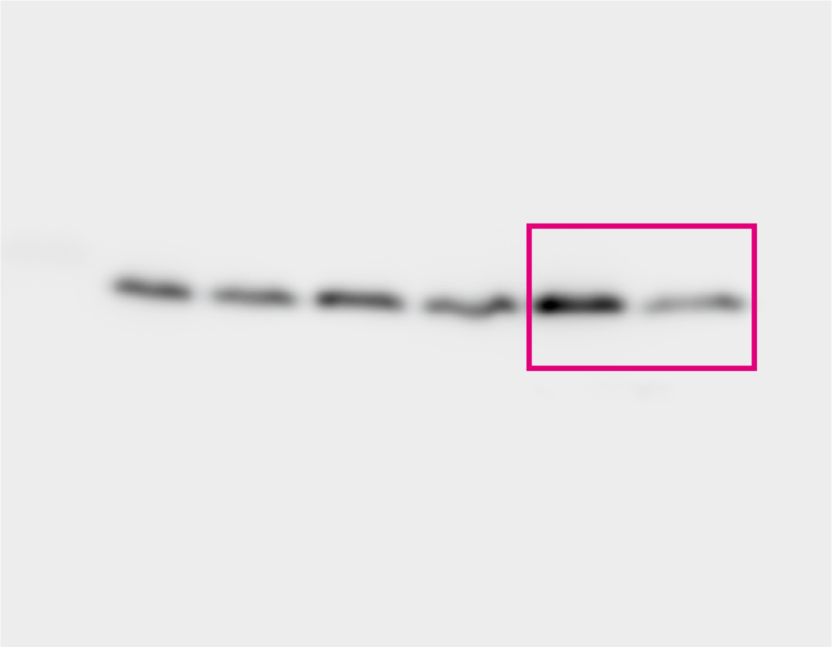

Figure 4 - Western blots (CDK2)

K562

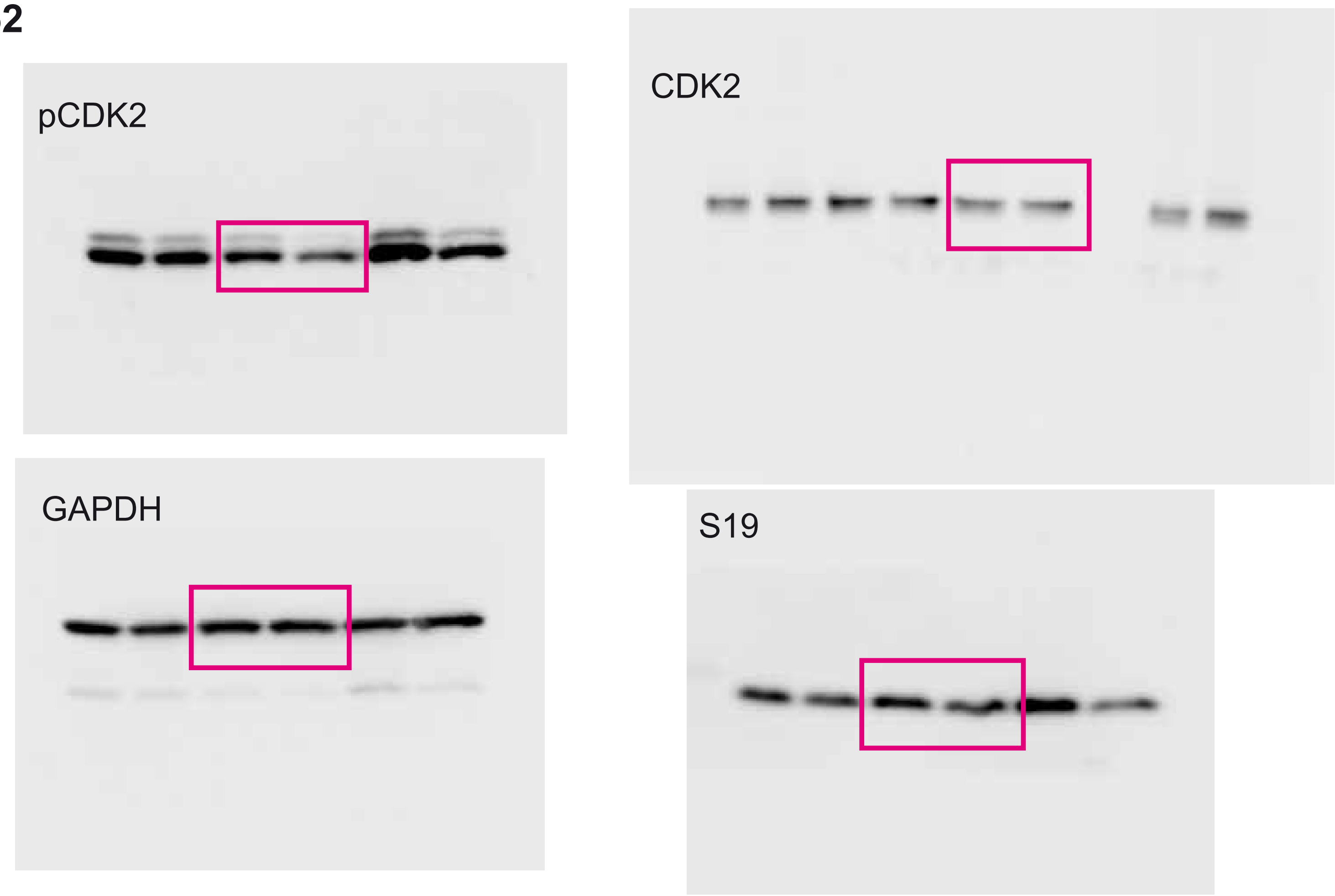

22Rv1

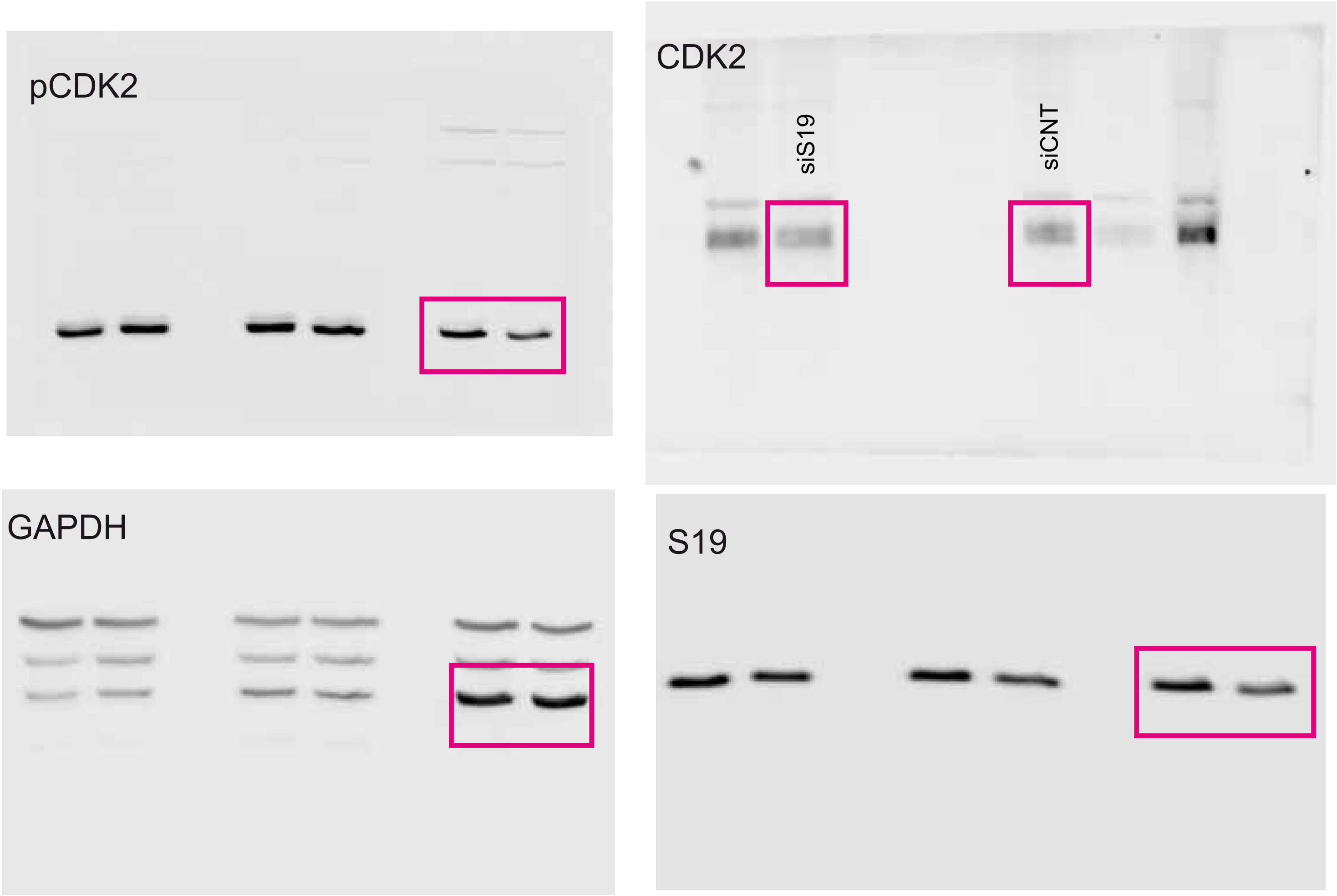

Figure 4 - Western blots (AKT, AMPK)

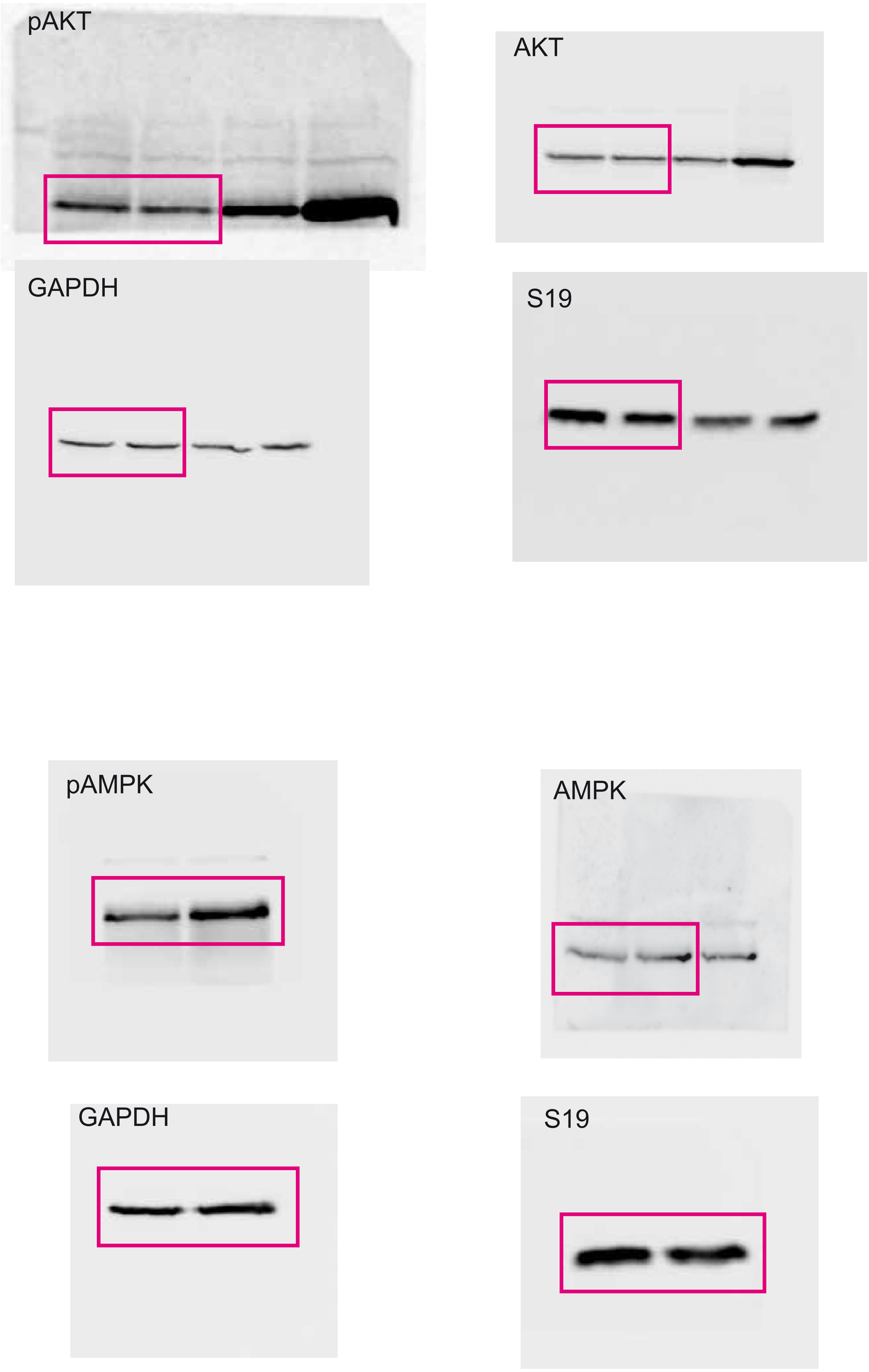

Supplement: Supplementary Information [file srep35026-s1.pdf]
